# Effectiveness Testing of a Piezoelectric Energy Harvester for an Automobile Wheel Using Stochastic Resonance

**DOI:** 10.3390/s16101727

**Published:** 2016-10-17

**Authors:** Yunshun Zhang, Rencheng Zheng, Keisuke Shimono, Tsutomu Kaizuka, Kimihiko Nakano

**Affiliations:** 1 Institute of Industrial Science, The University of Tokyo, 4-6-1, Komaba, Meguro, Tokyo 153-8505, Japan; topzrc@iis.u-tokyo.ac.jp (R.Z.); tkaizuka@iis.u-tokyo.ac.jp (T.K.); 2 Department of Mechanical Systems Engineering, Graduate School of Engineering, Tokyo University of Agriculture and Technology, 2-24-16, Naka-cho, Koganei-shi, Tokyo 184-8588, Japan; kshimono@go.tuat.ac.jp; 3 Interfaculty Initiative in Information Studies, The University of Tokyo, 4-6-1, Komaba, Meguro, Tokyo 153-8505, Japan; knakano@iis.u-tokyo.ac.jp

**Keywords:** wheel energy harvesting, piezoelectric, nonlinear bistable structure, stochastic resonance, tire pressure detection

## Abstract

The collection of clean power from ambient vibrations is considered a promising method for energy harvesting. For the case of wheel rotation, the present study investigates the effectiveness of a piezoelectric energy harvester, with the application of stochastic resonance to optimize the efficiency of energy harvesting. It is hypothesized that when the wheel rotates at variable speeds, the energy harvester is subjected to on-road noise as ambient excitations and a tangentially acting gravity force as a periodic modulation force, which can stimulate stochastic resonance. The energy harvester was miniaturized with a bistable cantilever structure, and the on-road noise was measured for the implementation of a vibrator in an experimental setting. A validation experiment revealed that the harvesting system was optimized to capture power that was approximately 12 times that captured under only on-road noise excitation and 50 times that captured under only the periodic gravity force. Moreover, the investigation of up-sweep excitations with increasing rotational frequency confirmed that stochastic resonance is effective in optimizing the performance of the energy harvester, with a certain bandwidth of vehicle speeds. An actual-vehicle experiment validates that the prototype harvester using stochastic resonance is capable of improving power generation performance for practical tire application.

## 1. Introduction

With the increasing requirements of advanced driving assistance systems, researchers have developed a tire-pressure monitoring system that can provide the driver with the tire condition via wireless transmission [1,2,3]. However, because it is inconvenient to replace or recharge the batteries of a monitoring system, there is a desire to establish an effective energy harvesting system [4]. The present paper therefore proposes a piezoelectric energy harvester for rotating automobile wheels, and focuses on experimental testing under on-road noise excitations to improve the harvesting efficiency through the application of stochastic resonance.

Several attempts have been made to exploit energy harvesters for rotating environments. Specifically, a linearly piezoelectric energy harvester using a cantilever beam has been proposed, where the centrifugal force is used to stimulate the natural frequency of the energy harvester system matching the rotational frequency [5]. A linear MEMS energy harvester using tire shock excitations was proposed for automotive applications; however, it can extract more energy only in the case of high-speed driving, and the energy harvesting efficiency depends on the concave-convex condition of the road surface [6]. A nanogenerator layer was attached on the inner surface of the tire, and the energy was harvested by the bending of the piezoelectric film; however, the energy harvesting performance was investigated using a bicycle tire instead of a real vehicle tire [7]. Moreover, a linear energy harvester was presented to optimize the frequency band of operation based on a feedback loop control system [8].

Nonlinear systems have a small structure, which can improve the performance over a wider bandwidth for harvesting more energy compared with a conventional linear system; therefore, they have also been used for rotational energy harvesting. An offset pendulum with a nonlinear bistable restoring spring structure has been developed to improve the operational bandwidth of the system considering the effects of gravity [9]. Additionally, a nonlinearly suspended energy harvester has been developed; its natural frequency varies because of variations in the transverse gravity force during wheel rotation [10]. However, the cited studies ignored the effect of road noise on the dynamic performances of the harvesting systems.

Meanwhile, stochastic resonance as a physical phenomenon has recently been proposed to explain the periodicity of Earth’s glaciation, where the output response can be greatly amplified with a certain probability by adding noise to a weak periodic signal [11]. In recent years, this phenomenon has attracted considerable attention for information transmission and detection [12,13,14,15], biological neuro analysis [16,17,18,19], and image processing [20,21,22,23]. In the mechanical engineering field, it has been theoretically investigated whether stochastic resonance can be applied to improve the efficiency of energy harvesting [24]. Consequently, an experimental study has realized the one-degree-of-freedom behavior of stochastic resonance in a bistable vibrating energy harvester [25]. Nevertheless, in the rotational environment of an automobile wheel, such behavior coexists with the two-degree-of-freedom motion of the wheel rotation and vibration. In a previous study, ideal white noise and a periodic force were employed to simulate rotational wheel motion, and the energy harvesting efficiency was improved by the realization of stochastic resonance [26]. By application of the on-road noise measured for low-speed automobile wheels, a mathematical model of vibration energy harvesting has been presented for the simulation analysis of stochastic resonance [27]. Furthermore, a macro-scale energy harvester has been fabricated, and only displacement response has been studied to know its dynamic performance [28]. However, it is still unclear for their harvesting effectiveness in a real-world environment.

Therefore, considering on-road noise and periodic acceleration by gravity for vehicle traveling on a paved road, the present study focuses on the effectiveness testing of a piezoelectric energy harvester on vehicle tires using stochastic resonance, and the energy harvesting characteristics are comprehensively investigated through simulation and laboratory experimental analyses. It shows that the appearance of stochastic resonance is not limited to a constant rotation frequency but can be maintained under varying frequencies, indicating that more energy can be harvested with a bandwidth for rotational systems. Finally, the actual-vehicle experiment is implemented for the validation of stochastic resonance in real tire application.

The paper is organized as follows: Section 2 elaborates on the mathematic model of the energy harvester. Section 3 describes the experimental study in terms of the experimental setup, on-road noise measurement, and its processing. Section 4 presents numerical simulation and experiment results, and then discussions are given in Section 5, while Section 6 draws conclusions from the results of the study.

## 2. Mathematical Model

**Figure 1:**
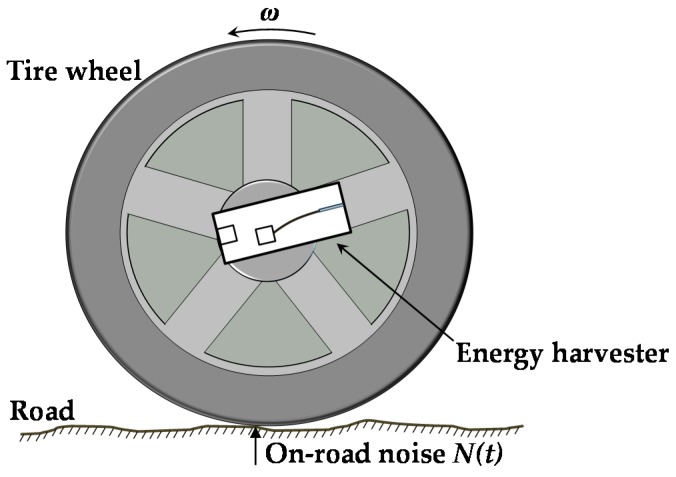
Illustration of the energy harvester attached to the center of a wheel.

Figure 1 shows that a macro-scale energy harvester can be attached to a wheel rotating counterclockwise with angular velocity *ω*. The vertical on-road noise *N*(*t*) is produced by the interaction of the rotating tire and the road surface.

**Figure 2:**
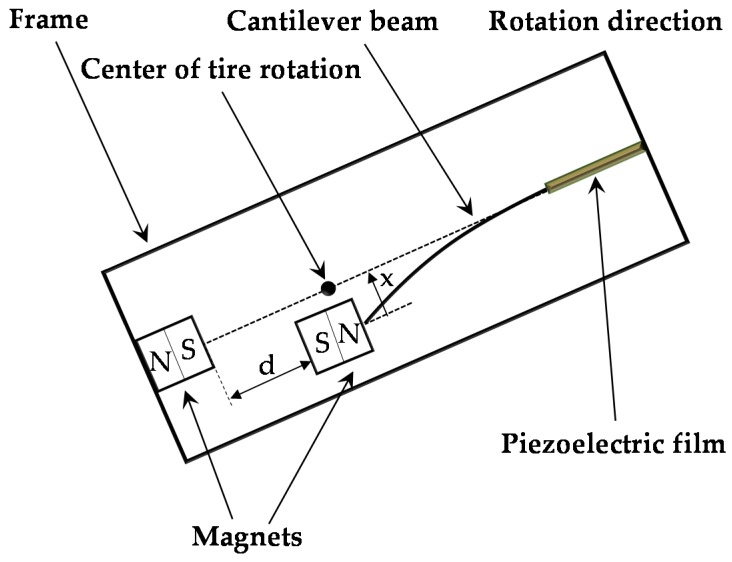
Diagram of the nonlinear bistable energy harvesting configuration.

To eliminate the effect of the centrifugal forces, the center of the cantilever tip mass is located at the rotational center of the wheel due to the sensitivity of cantilever beam stiffness to centrifugal force, as shown in Figure 2. To obtain the restoring force between the two magnets of the energy harvester, a mathematical model is first derived for the interaction forces of the magnets. The dipole model is used to represent the interaction forces, and the potential energy of the movable tip magnet can be defined as

(1) $$U_{M}=-m_{c}\cdot B$$

where **m***_c_* is the magnetic dipole moment vector of the fixed permanent magnet, defined by **m***_c_* = **M**_c_ *v*. **B** is the magnetic flux density, **M***_c_* = (*M_cx_*, *M_cy_*) denotes the magnetization strength of the permanent magnet bonded to the frame in terms of its vertical and horizontal components, and *v* is the volume of the magnets.

Then, the potential energy of the magnetic end mass is expressed as

(2) $$U_{M}=\frac{\mu_{0}v^{2}}{4\pi }\Phi (x)$$

where *μ*_0_ is the permeability of free space and *x* is the vibrational displacement of the end magnet. From reference [29], the function Ф(*x*) is defined as

(3) $$\Phi (x)=-\frac{3\left(dM_{cy}+xM_{cx}\right)\left(dM_{fy}+xM_{fx}\right)}{{\left(d^{2}+x^{2}\right)}^{5/2}}+\frac{M_{fy}M_{cy}+M_{fx}M_{cx}}{{\left(d^{2}+x^{2}\right)}^{3/2}}$$

where *d* is the lateral separation of the two magnets. Note that **M***_f_* = (*M_fx_*, *M_fy_*) denotes the magnetization strength of the magnetic tip mass in terms of its vertical and horizontal components; the two magnets should have opposite polarity, indicating repulsion.

The restoring forces are obtained from the spatial derivative of the potential energy as

(4) $$F_{M}(x)=\frac{\mu_{0}v^{2}}{4\pi }\Theta (x)$$

where Θ(*x*) is expressed as

(5) $$\Theta (x)=\frac{3\left[M_{cy}\left(dM_{fx}+xM_{fy}\right)+M_{cx}\left(dM_{fy}+3xM_{fx}\right)\right]}{{\left(d^{2}+x^{2}\right)}^{5/2}}-\frac{15x\left(xM_{fx}+dM_{fy}\right)\left(xM_{cx}+dM_{cy}\right)}{{\left(d^{2}+x^{2}\right)}^{7/2}}$$

**Figure 3:**
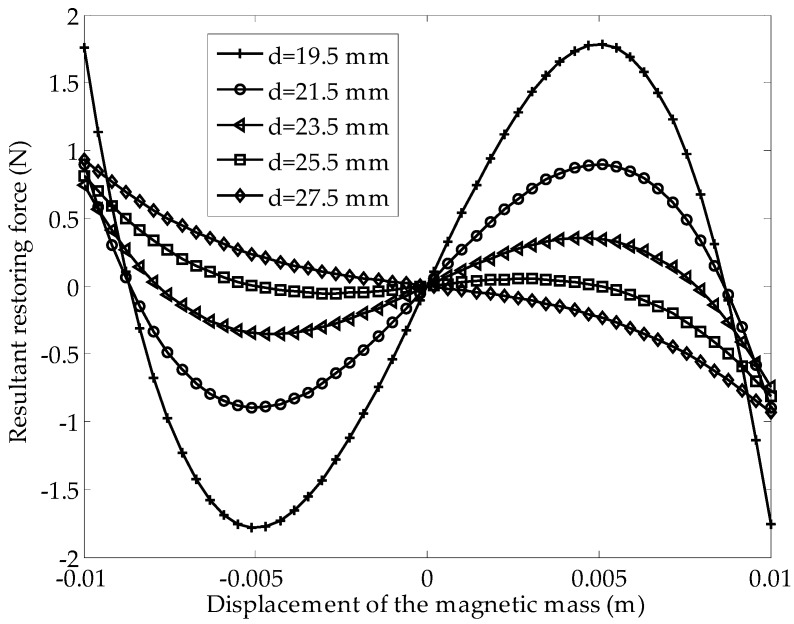
Resultant force versus the distance between the two magnets.

By substituting Equation (5) into (4), the restoring forces of the magnetic end mass can be derived, and the resultant restoring force of the beam is expressed as follows

(6) $$F(x)=F_{M}(x)-kx$$

where *k* is the spring constant of the beam. As shown in Figure 3, with the distance between the two magnets increasing from 19.5 to 25.5 mm, there are two stable states, and the distance between the two stable points gradually becomes small. When the separation of the magnets exceeds 25.5 mm, the cantilever beam cannot be stimulated as being bistable, and it becomes a monostable system around the equilibrium position. Table 1 gives the numerical parameters used to investigate the effect of the distance between the two magnets on the steady state of the system.

Equation (6) can be further expressed in Taylor series, calculated around *x* = 0 and simplified as follows

(7) $$F(x)=\left(\mu_{0}v^{2}\frac{9M_{cx}M_{fx}-12M_{cy}M_{fy}}{4\pi d^{5}}-k\right)x-\left(\mu_{0}v^{2}\frac{75M_{cx}M_{fx}-90M_{cy}M_{fy}}{8\pi d^{7}}\right)x^{3}$$

Equation (8) shows the Duffing dynamic equation of the wheel rotation

(8) $$m\ddot{x}+c\dot{x}-ax+bx^{3}=N(t)+G\sin\left(\omega t+\theta_{0}\right)$$

where *m* is the mass of the tip magnet, *c* is the viscous damping coefficient of the beam, and *N*(*t*) is the noise excitation from the road surface. With anticlockwise rotation of the wheel with angular velocity *ω* from an initial angle *θ*_0_, the effect of gravity on the end magnet produces periodic gravity force, which is expressed as *G*sin(*ωt*). In this, the *a* shown in the Equation (8) is the combinational linear coefficient of beam and magnets, and *b* is the nonlinear coefficient of the relationship between the two magnets.

Thus, the resultant restoring force of beam can be given as

(9) $${F_{M}}^{\prime }=ax-bx^{3}$$

Due to the relation of *F_M_’* = *F*(*x*), Equations (10) and (11) are derived by employing the corresponding constant terms of Equations (7) and (9), respectively.

(10) $$a=\mu_{0}v^{2}\left(\frac{9M_{cx}M_{fx}-12M_{cy}M_{fy}}{4\pi d^{5}}\right)-k$$

(11) $$b=\frac{\mu_{0}v^{2}\left(75M_{cx}M_{fx}-90M_{cy}M_{fy}\right)}{8\pi d^{7}}$$

In the case of weak friction, the Kramers rate can be modified from reference [30] as

(12) $$\omega_{SR}<\frac{\omega_{0}}{2}\exp\left(-\frac{a^{2}}{4bD}\right)$$

By substituting Equations (10) and (11) into (12), the Kramers rate can be presented as a function of the distance between the two magnets *d*. Here, *ω_SR_* is the angular velocity of the modulation force, *ω*_0_ = (2*a*/*m*)^1/2^ is the resonance angular velocity of the bistable system, and *D* is the intensity of the noise excitation from the road surface.

## 3. Experimental Study

### 3.1. Experimental Setup

**Figure 4:**
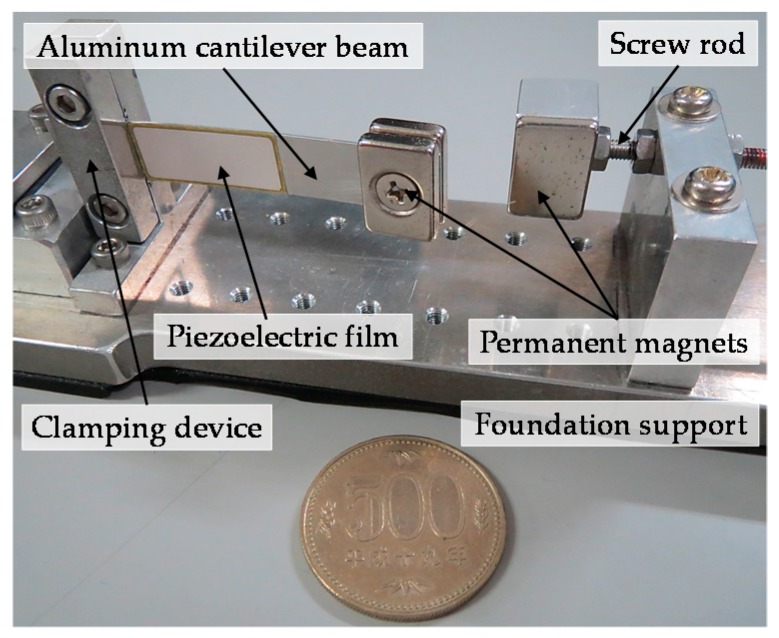
Diagram of the macro-scale energy harvester (The coin with diameter of 26.5 mm).

**Figure 5:**
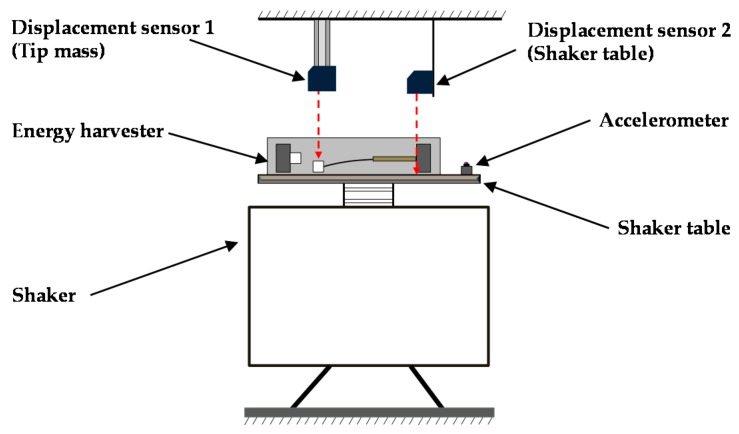
Schematic diagram of the experimental setup.

**Figure 6:**
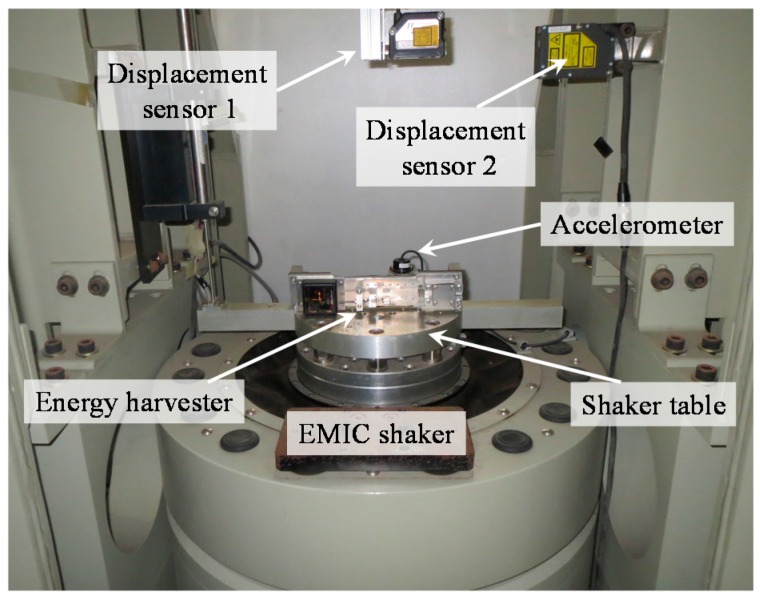
Overview setting of experiment apparatus.

The energy harvester consists of a 42 mm × 10 mm × 4 mm aluminum cantilever beam and an adjustable screw rod for adjusting the distance between two magnets. The magnetic tip mass comprises two identical neodymium magnets (NSC0010, Miyagi, Japan) with dimensions of 15 mm × 10 mm × 3 mm and a permanent magnet (NS0093, Miyagi, Japan) with dimensions of 15 mm × 10 mm × 2 mm. Ultrathin unimorph ceramic piezoelectric material (K2512U1, THRIVE Corporation, Saitama, Japan) with dimensions of 22.9 mm × 10 mm × 0.1 mm was selected to paste on the root segment of the beam, and its electrostatic capacitance is 115 nF. The N/S pole of the magnets is still kept in the *x*-axis, which is the same arrangement with the model in Figure 2. The piezoelectric layer can only bend along the thickness direction of the cantilever, which maximizes the stress on the piezoelectric layer and thus improves power generation. The volume of the energy harvester is 15.75 cm^3^ (70 mm × 15 mm × 15 mm), as shown in Figure 4. Figure 5 presents the experimental setup. The energy harvester system is fixed on the shaker table, displacement sensors 1 and 2 are set to measure the vibrational amplitudes of the magnetic end mass and shaker table, respectively, and the acceleration sensor is assembled to detect the vibration acceleration of the shaker table. An overview of the experimental setting is shown in Figure 6. A shaker (F-08000BDH/SLS16/Z02, EMIC Corporation, Tokyo, Japan) is used to generate periodic and noise vibration with peak-to-peak amplitude up to 50 mm. In addition, the response frequency with a range of 0–2000 Hz can act as the external input for wheel rotation.

### 3.2. On-Road Noise Measurement

**Figure 7:**
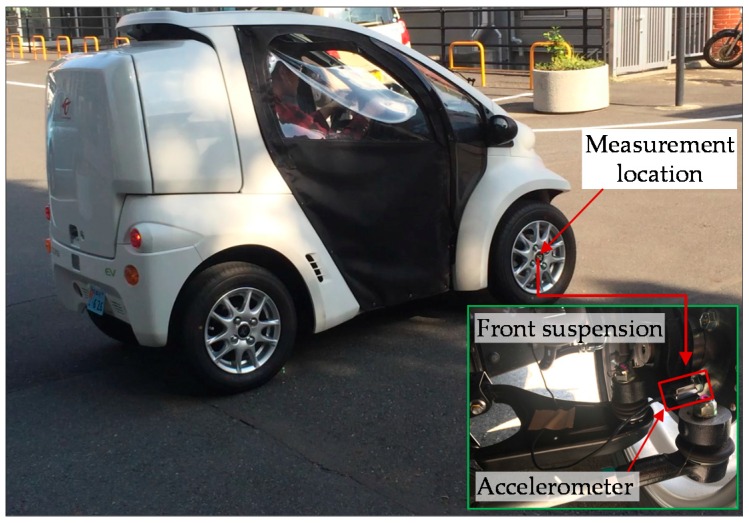
Paved road testing and on-road noise measurement on front-wheel suspension.

The experimental environment shown in Figure 7 features a smooth paved road and electric vehicle (Coms, ZAD-TAK30-DS, Toyota Auto Body Co., Ltd., Aichi, Japan). To measure the road noise, a wireless acceleration sensor (MVP-RF3-J, MicroStone Corporation, Nagano, Japan) with a response frequency of 1–1000 Hz is installed on the front suspension of the vehicle. In low speed range conditions where the vehicle travels on a smooth paved road, it indicates that the power spectral density of the accelerations remains identical by analyses of the measured signal. Therefore, the measured acceleration under 20 km/h is selected as the input condition for simulation study, and then the intensity of the on-road noise can be derived. Calculation of the Kramers rate reveals that stochastic resonance readily occurs at an angular velocity lower than 40.6 rad/s (6.5 Hz).

### 3.3. On-Road Noise Processing

**Figure 8:**
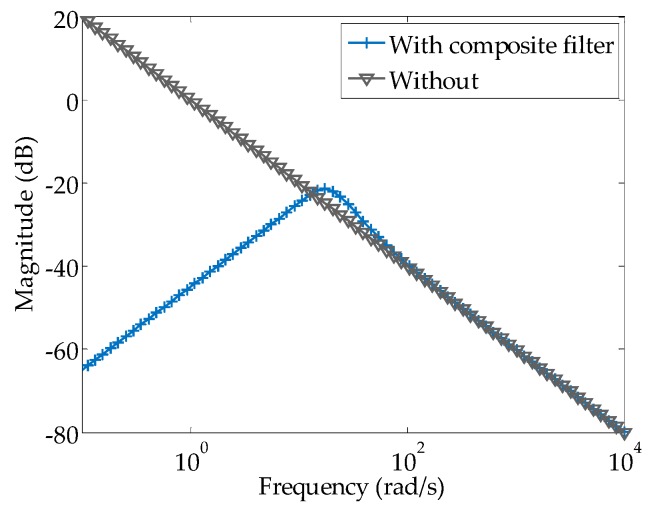
Comparison of the frequency responses of the traditional integration method and composite filter.

Because the shaker provides the velocity control input, integration of the measured road surface acceleration signal into the velocity signal is essential for the accurate regeneration of the on-road noise excitation. Simultaneously, to eliminate the nondetectable low-frequency effect below 1 Hz, with reference to the literature [31], a composite filter is designed; the transfer function *G*(*s*) is derived as

(13) $$G\left(s\right)=\frac{s^{2}+2\omega_{cc}s}{s^{3}+2\omega_{cc}s^{2}+2{\omega_{cc}}^{2}s+{\omega_{cc}}^{3}}$$

where *ω_cc_* is the determination coefficient for eliminating the effect of the low-frequency region. This is done instead of employing the traditional integral method, which can produce accumulative errors, as shown in Figure 8.

By using this kind of filter, the acceleration signal is calculated above 1 Hz as

(14) $$f(t)=\left\{ \begin{array}{l} t,\;\left(0\leq t\leq 1\right)\\ 1,\;\left(1<t\leq \gamma \right) \end{array}\right.,\;g(t)=\left\{ \begin{array}{l} 1,\;\left(0\leq t\leq \gamma -1\right)\\ t,\;\left(\gamma -1<t\leq \gamma \right) \end{array}\right.$$

Here, *t* and γ are respectively the real time and total experiment time, *f*(*t*) is the linear amplification for gradually increasing the vibrational amplitude of the shaker within 1 s of the start time, and *g*(*t*) is the linear attenuation for weakening the vibration in the final second before the shaker completely stops shaking.

**Figure 9:**
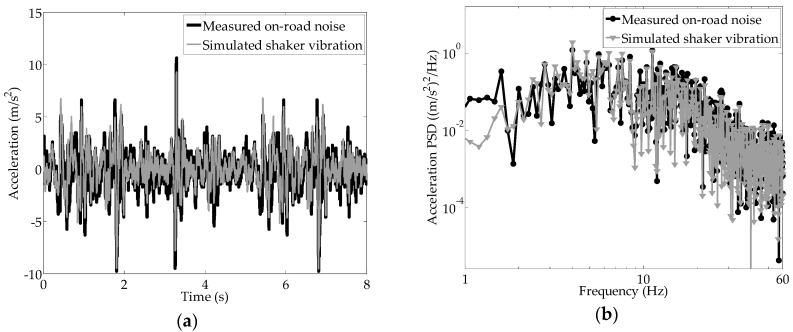
(**a**) Acceleration regeneration of the measured on-road noise for the shaker; (**b**) power spectrum density comparison of the measured on-road noise and simulated shaker vibration.

When the vehicle travels at low speed, with the purpose of regenerating the on-road noise indicated by the black solid line in Figure 9a, the power spectral density of the measured on-road acceleration is further analyzed in Figure 9b [28]; it shows that the energy from paved road noise excitation is mainly restricted to the frequency bandwidth of 1–60 Hz while the vehicle travels at low speed. In Figure 9a,b, the simulated vibration from the shaker is approximately consistent with the on-road noise measured using a composite filter.

## 4. Results

### 4.1. Numerical Simulation

**Figure 10:**
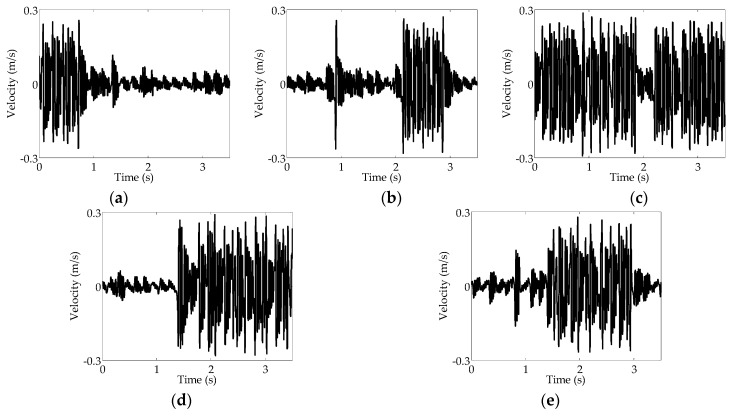
Velocity responses under the on-road noise excitation and the periodic gravity force at the angular frequencies: (**a**) 33 rad/s; (**b**) 35 rad/s; (**c**) 38 rad/s; (**d**) 40 rad/s; (**e**) 42 rad/s.

The numerical simulation was implemented under the conditions of periodic gravity force and the measured on-road noise excitation, and the related results are depicted in Figure 10. The angular velocity of the rotational tire is varied from 33 rad/s to 42 rad/s.

As shown in Figure 10a, when the angular velocity is 33 rad/s, the velocity response of the cantilever beam remains at a high level for a short initial time; however, the cantilever beam subsequently fluctuates around a low velocity, which illustrates that the beam vibrates within one potential well. Figure 10b shows that there is more inter-well motion of the cantilever beam at an angular velocity of 35 rad/s than at 33 rad/s. Figure 10c shows that when *ω* is 38 rad/s, the velocity responses of the cantilever beam maintain a high-velocity motion. The probability of the beam jumping between two stable positions thus becomes higher as angular velocity increases. Figure 10d,e show that, beyond 38 rad/s, even the velocity responses of the cantilever beam become low, and inter-well motion can still be induced at shorter time periods.

Thus, when the angular velocity of the rotating wheel is lower than 42 rad/s, the response of the system is enhanced by the appearance of inter-well motion. At an angular velocity of 38 rad/s, stochastic resonance is easily stimulated compared with the other four cases, which indicates that the occurrence of stochastic resonance at the angular velocity of 38 rad/s practically agrees with the result of the theoretical analysis of 40.6 rad/s as calculated in Section 3.2.

### 4.2. Laboratory Test Result

The first experiment investigated the dynamic performance of the energy harvester and the collected power with a matched resistive load. The second experiment validated the sustainability of the energy harvester under a linearly increasing frequency of up-sweep excitations with constant amplitude of gravitational acceleration. The primary goal of the laboratory experiment is to verify the dynamic performance of the prototype system under three conditions: (1) on-road noise excitation; (2) periodic gravity force; and (3) wheel rotation.

**Figure 11:**
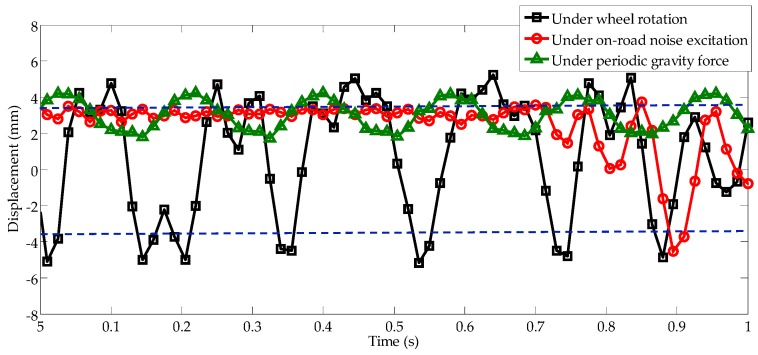
Displacement responses under three conditions: (1) on-road noise excitation; (2) periodic gravity force; and (3) wheel rotation combined with the on-road noise excitation and the periodic gravity force.

As shown in Figure 11, the displacement responses can be observed under the above mentioned three conditions, and the two stable equilibrium positions are located at ±3.5 mm, as indicated by blue dashed lines. First, under the condition of on-road noise excitation, by suitably separating the magnets, the displacement response of the system remains at a low level, and the cantilever beam occasionally stimulates escape from one stable position to another stable position, depending on the degree of bumps in the paved road. Second, under periodic gravity force with modulation frequency of 6 Hz, the cantilever beam vibrates at a position between 2 and 4 mm with intra-well dynamic oscillation. Third, under wheel rotation, the energy harvester experiences not only excitations from its own periodic gravity force but also on-road noise excitation. The black line in Figure 11 shows that the system enters inter-well motion between two stable equilibrium positions, and the displacement response is strongly amplified at a frequency of 6 Hz, which matches the simulation parameters calculated using the Kramers rate. This demonstrates that the occurrence of stochastic resonance is feasible for real applications.

**Figure 12:**
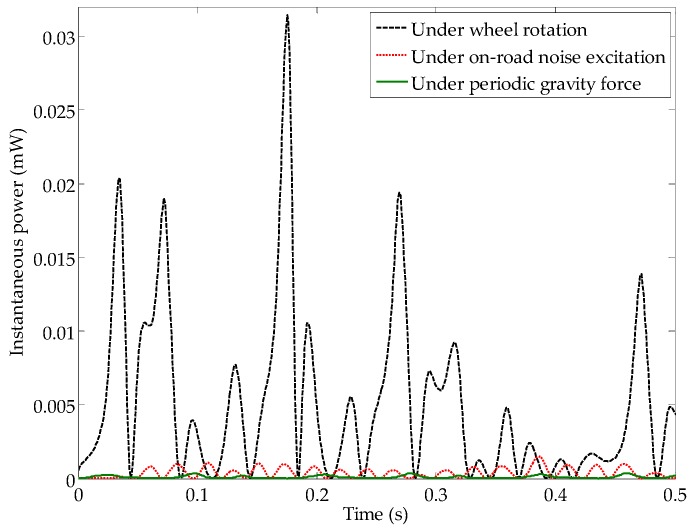
Comparison of the available net power under three conditions.

The load resistance experiment was carried out with a matching resistor of 252 kΩ to confirm performance improvement due to the occurrence of stochastic resonance under real circumstances. Figure 12 shows that the harvesting system can capture a maximum power of 0.032 mW, and an average power of 0.0063 mW by using root mean square (RMS) voltage with wheel rotation. The average power is approximately 12 times that under the condition of on-road noise excitation and 50 times that under the effect of the periodic gravity force.

**Figure 13:**
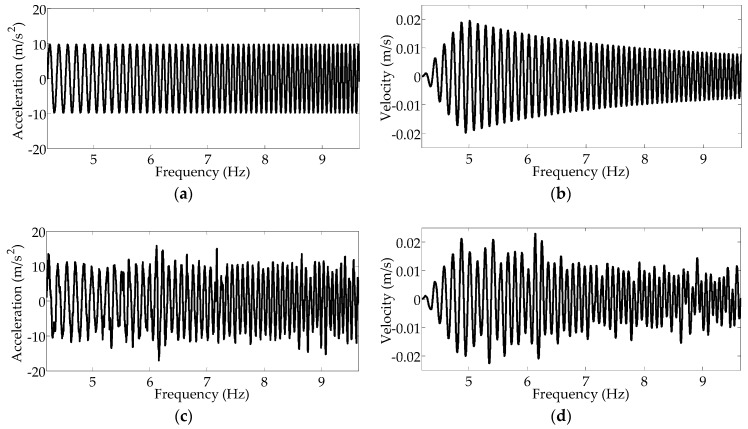
(**a**) Up-sweep excitation curve for gravitational acceleration; (**b**) up-sweep excitation curve for the corresponding velocity; (**c**) up-sweep excitation curve for gravitational acceleration in conjunction with the simulated on-road noise excitation; (**d**) up-sweep excitation curve for the corresponding velocity in conjunction with the simulated on-road noise excitation.

In the second experiment, an increasing up-sweep frequency was adopted for validation of the sustainability of stochastic resonance. Considering the amplitude limitation of the shaker and gravitational acceleration of 9.8 m/s^2^, the frequency of change in acceleration and velocity was linearly increased from 4.2 to 9.7 Hz within 6.5 s. Corresponding to a vehicle speed range of 27–63 km/h, the increasing sweep frequency of the acceleration and velocity are presented in Figure 13a,b, respectively; moreover, for the case where the on-road noise excitation is interfused, the increasing sweep frequencies of the acceleration and velocity are presented in Figure 13c,d, respectively.

**Figure 14:**
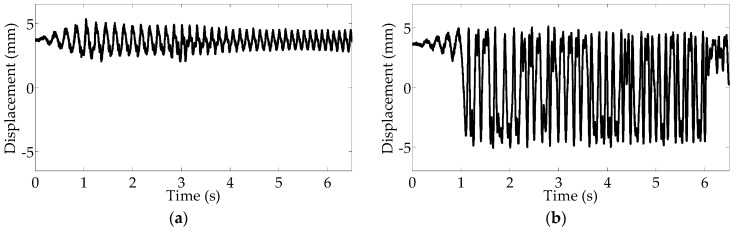
Experimental displacements: (**a**) under the up-sweep excitation; (**b**) under the combination of the up-sweep excitation and on-road noise excitation.

In Figure 14a, under only the up-sweep excitation with gravitational acceleration of 9.8 m/s^2^, the displacement response of the cantilever beam keeps the lower vibration level at one stable position throughout the frequency range. However, Figure 14b shows that when the up-sweep excitation and road noise are input simultaneously, the intake energy is increased to drive the high displacement response to the occurrence of stochastic resonance, owing to the presence of the additional ambient noise excitation. The vibration response is therefore enhanced with the inter-well dynamic oscillation, and even the up-sweep frequency varies during the period of 1–6 s which corresponds to that of the frequencies of 5–9.2 Hz. This demonstrates that the occurrence of stochastic resonance is not limited to a certain frequency, but can be maintained within a certain frequency bandwidth of 5–9.2 Hz.

**Figure 15:**
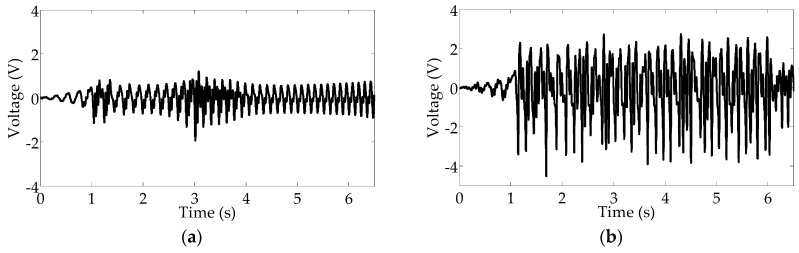
Produced voltages: (**a**) under up-sweep excitation; (**b**) under the combination of up-sweep excitation and on-road noise excitation.

**Figure 16:**
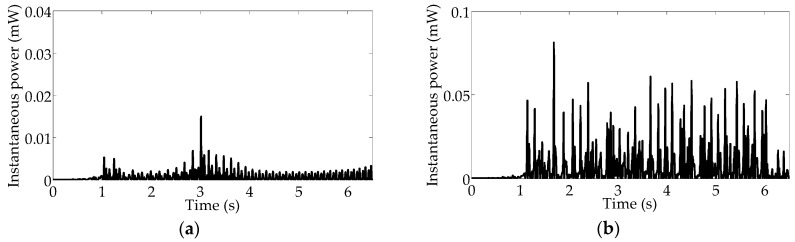
Harvested powers: (**a**) under up-sweep excitation; (**b**) under the combination of up-sweep excitation and on-road noise excitation.

Figure 15 presents the voltages harvested at different rotational frequencies. The voltage reaches a maximum value of 1.2 V with a RMS value of 0.4 V, in Figure 15a; however, when the road noise is added to the separate periodic signal, the maximum voltage reaches 4.5 V with a higher RMS value of 1.7 V, in Figure 15b. Correspondingly, as shown in Figure 16b, the maximum power is 0.081 mW with average power of 0.012 mW, and the power density is obtained as 0.76 μW/cm^3^ by calculating the average value of power per unit volume for the energy harvester. Apparently, the power is improved with combined excitation, and the harvesting system can be activated to high-energy motion owing to the occurrence of stochastic resonance. Importantly, the high-energy motion can be effectively maintained with the same varying rotational frequencies of 5–9.2 Hz. Consequently, the results of the experimental testing demonstrate that stochastic resonance is capable of enhancing energy harvesting over a wide range of vehicle speeds.

### 4.3. Actual-Vehicle Experiment

**Figure 17:**
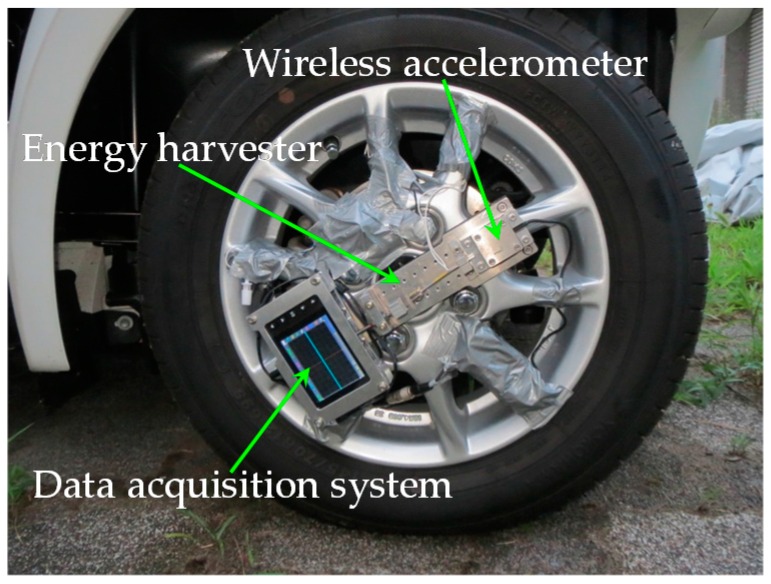
Apparatus for the actual-vehicle experiment.

As shown in the Figure 17, the proposed energy harvester is attached to the front tire wheel of the electric vehicle to verify the power generation performance in real-world road environment. The center of the cantilever tip mass is precisely located at the rotational center of the wheel to eliminate the centrifugal force’s effect on the balance of the wheel and stiffness of the cantilever beam. The wireless accelerometer is mounted on the wheel for measuring the on-road noise excitation and tangential gravity acceleration, and a data acquisition system (DSO Nano v3, SeeedStudio, Shenzhen, China) is used to continuously measure real-time output voltage across a matched load resistor.

**Figure 18:**
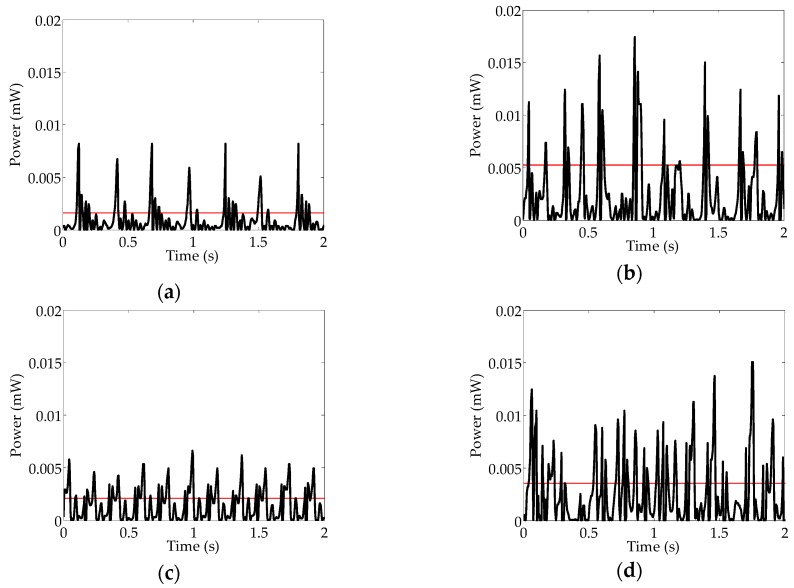
Power generation corresponding to different speeds: (**a**) 10 km/h; (**b**) 20 km/h; (**c**) 30 km/h; (**d**) 40 km/h.

**Figure 19:**
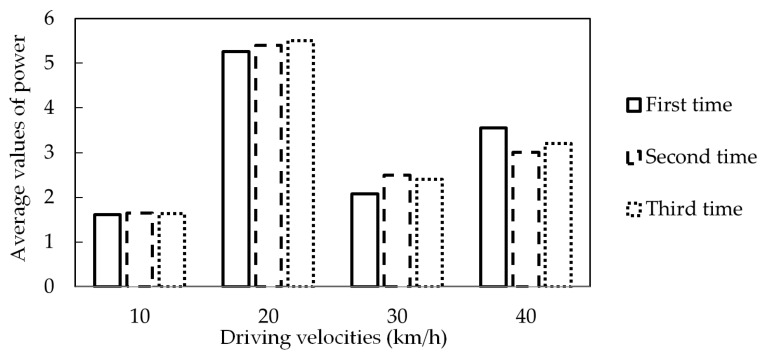
Average values of the collected power under four different speeds.

It should be noted that when the energy harvester is rotating as the vehicle drives, considering the dynamic effect of rotation on the on-road noise that is forced on the cantilever, the expression of the on-road noise becomes *N*(*t*)*sinωt* instead of *N*(*t*). Then the intensity of *N*(*t*) is weakened to stimulate the phenomenon of stochastic resonance at an angular velocity lower than 22.5 rad/s (23.5 km/h). The validation experiment is implemented on real-world road, in which the vehicle travels at four different speeds of 10 km/h, 20 km/h, 30 km/h, and 40 km/h. As presented in Figure 18, the power generation performances are given under four different vehicle speeds, and the red solid line represents the average value of power. The experimental results indicate that the power can be boosted to a higher level at the speed of 20 km/h, due to the occurrence of stochastic resonance. It is approximated to the calculated Kramers rate value of 23.5 km/h. As presented in Figure 18c,d for the increasing vehicle speeds of the 30 km/h and 40 km/h, it becomes a tendency that the higher tire rotating angular velocity and on-road noise intensity resulted in higher collected power; nevertheless, the power generation levels are still lower than that of the 20 km/h for the case of stochastic resonance. Furthermore, as presented in Figure 19, it is indicated that the average values of the collected power can be obviously enhanced at the velocity of 20 km/h compared to the other three different driving conditions. Meanwhile, this tendency is also validated by implementing the test three times for every speed.

## 5. Discussions

The low power density mainly resulted from the application of the piezoelectric material. In the experimental study, a unimorph ultrathin ceramic type of the piezoelectric material was applied with small dimensions of 22.9 mm × 10 mm × 0.1 mm. Moreover, when the matched load resistance of the piezoelectric material becomes high, the harvested power is decreased because the piezoelectric layer has a small electrostatic capacitance of 115 nF. In addition, it should be noted that the prototype of the energy harvester has to include a wireless motion recorder, accelerometer, data acquisition system, and the foundation support, to accomplish whole experimental procedure.

Therefore, it is considered that the output power can be improved by enlarging the size of piezoelectric films, utilizing a bimorph type of piezoelectric films instead of a unimroph one, and increasing the value of the piezoelectric electrostatic capacitance to decrease the matched load resistance. On the other hand, it is also worth considering that multiple cantilevers can be used to increase energy harvesting.

In the actual-vehicle experiment, it became difficult to maintain the steady velocities at the required increment of 5 km/h. Therefore, to ensure testing accuracy and better control, the driving speed was implemented for every 10 km/h. This study aims at realizing stochastic resonance to improve the performance of an energy harvester, which is attached on rotating tire around low speed range. The maximum velocity of 40 km/h is sufficient to satisfy experimental conditions, to validate whether the phenomenon can be observed at the actual-vehicle tire. Additionally, when the driving experiment was processed in campus, the actual-vehicle driving velocity is limited to 40 km/h. However, it is an interesting topic to test the energy harvesting performance in a wider range and smaller interval of driving speed.

This study attempted to improve the performance of energy harvesting by stimulus of the stochastic resonance, in which the energy harvester is mounted in the center of the tire wheel. In this case, cable transmission can be used to power the TPMS. If the energy harvester is installed inside the tire, the effect of centrifugal force on the stiffness of the cantilever may influence the stimulus of stochastic resonance. However, it is still possible to exploit stochastic resonance, by adjusting the related parameters of the energy harvesting system for reducing the initial spring constant. Therefore, it is about how to realize stochastic resonance while considering more external factors’ influence on the energy harvesting system. It can be considered as a challenging future work.

## 6. Conclusions

The present study applied the periodic gravity force and on-road noise excitation of the rotating wheel to validate the effectiveness of energy harvesting by the application of the phenomenon of stochastic resonance. The systematic structure was modeled under the real condition of on-road noise excitation, when a vehicle travels on a smooth paved road. The measured on-road noise was processed using a composite filter to provide the practical condition, which is suitable for experimental up-sweep explorations.

By implementing a laboratory test, it was found that stochastic resonance can improve the energy harvesting performance of a rotating wheel. The experimental results revealed inter-well motion at a frequency of 6 Hz for wheel rotation, where the captured power was 12 times that under the condition of only on-road noise excitation and 50 times that under the condition of only the periodic gravity force. More importantly, it was validated that as the periodic frequency varies, the phenomenon of stochastic resonance is sustainable in terms of optimizing the performance of the energy harvester with a power density of 0.76 μW/cm^3^. This demonstrates that stochastic resonance is not limited to occurring at a constant rotational frequency but can be maintained under changing frequencies of 5–9.2 Hz.

Finally, the actual-vehicle experiment validates that the presented energy harvester is capable of triggering stochastic resonance for enhancing power generation performance. The approach is thus effective in absorbing higher energy for the application of tire pressure detection.

## Figures and Tables

**Table 1:** Parameters of the energy harvester.

Items	Permeability of Free Space	Volume of Magnet	Tip Mass	Magnetization Amplitude		Coefficient
Parameter	*μ*_0_	*v*	*m*	*M_fx_*, *M_fy_*	*M_cx_*, *M_cy_*	*k*
Value	4*π* × 10^7^ H/m	1.07 × 10^-6^ m^3^	8 g	–9 × 10^5^ A/m	8.5 × 10^5^A/m	152.7 N/m
